# Ciprofloxacin induces apoptosis and inhibits proliferation of human colorectal carcinoma cells

**DOI:** 10.1038/sj.bjc.6600079

**Published:** 2002-02-01

**Authors:** C Herold, M Ocker, M Ganslmayer, H Gerauer, E G Hahn, D Schuppan

**Affiliations:** Department of Medicine I, University of Erlangen-Nuernberg, Krankenhausstr. 12, D-91054 Erlangen, Germany

**Keywords:** apoptosis, cell cycle, ciprofloxacin, colorectal cancer, proliferation, caspase

## Abstract

Efficacy of chemotherapy in advanced stages of colorectal tumours is limited. The quinolone antibiotic ciprofloxacin was recently shown to inhibit growth and to induce apoptosis in human bladder carcinomas cells. We investigated the effect of ciprofloxacin on colon carcinoma lines *in vitro*. CC-531, SW-403 and HT-29 colon carcinoma and HepG2 hepatoma cells (control cells) were exposed to ciprofloxacin. Proliferation was assessed by bromodeoxyuridine-incorporation into DNA and apoptosis was measured by flow cytometry after propidium iodide or JC-1 staining. Expression of anti-apoptotic Bcl-2 and pro-apoptotic Bax was analyzed by semiquantitative Western blot analysis and activity of caspases 3, 8 and 9 by substrate-cleavage assays. Ciprofloxacin suppressed DNA synthesis of all colon carcinoma cells time- and dose-dependently, whereas the hepatoma cells remained unaffected. Apoptosis reached its maximum between 200 and 500 μg ml^−1^. This was accompanied by an upregulation of Bax and of the activity of caspases 3, 8 and 9, and paralleled by a decrease of the mitochondrial membrane potential. Ciprofloxacin decreases proliferation and induces apoptosis of colon carcinoma cells, possibly in part by blocking mitochondrial DNA synthesis. Therefore, qualification of ciprofloxacin as adjunctive agent for colorectal cancer should be evaluated.

*British Journal of Cancer* (2002) **86**, 443–448. DOI: 10.1038/sj/bjc/6600079 www.bjcancer.com

© 2002 The Cancer Research Campaign

## 

Cancers of the colon and rectum are the most common gastrointestinal neoplasms, with an incidence of about 44 per 100 000 per year (Boyle and Langman, 2000; Ries *et al*, 2000). Prognosis and therapy are determined by tumour stage, as reflected by the TNM- or the Dukes-classification, the involvement of lymph nodes and the presence of metastases. Operated patients with lesions restricted to the mucosa and submucosa (Dukes A or T_1_N_0_M_0_) have a 90% 5-year-survival, whereas prognosis of patients with advanced stages of the disease (Dukes D or T_1_N_0_M_0_) is poor. The first-line treatment is radical surgical resection with local lymph node dissection. In advanced stages of the disease, the effect of palliative chemotherapy is limited (Midgley and Kerr, 1999; Young and Rea, 2000).

It was recently shown that the fluoroquinolone ciprofloxacin, a commonly used broad-spectrum antibiotic with low side effects, can induce time- and dose-dependent growth inhibition and apoptosis of bladder carcinoma (Seay *et al*, 1996; Ebisuno *et al*, 1997; Aranha *et al*, 2000), osteosarcoma (Miclau *et al*, 1998) and leukaemia cell lines (Some *et al*, 1989). Fluoroquinolone antibiotics inhibit the bacterial type II topoisomerase/DNA gyrase which is responsible for supercoiling, transcription, replication and chromosomal separation of prokaryotic DNA (Chen and Liu, 1994). How fluoroquinolone antibiotics might affect mammalian cells is still unclear. There are no data on the effects of fluoroquinolone antibiotics on human colon carcinoma cell proliferation and apoptosis. We therefore investigated the anti-proliferative and pro-apoptotic effects of ciprofloxacin on the colon carcinoma cell lines CC-531, SW-403 and HT-29.

## MATERIALS AND METHODS

### Cell culture

CC-531 cells (rat, colorectal cancer, established from a thioacetamide-induced colon carcinoma) were cultured in RPMI 1640 medium (Biochrom, Berlin, Germany) with 10% foetal calf serum (FCS) (Gibco–BRL, Karlsruhe, Germany), penicillin (100 U l^−1^), streptomycin (10 mg l^−1^) and ascorbic acid (50 mg l^−1^) at 37°C and 5% CO_2_. HT-29 cells (human colorectal cancer, well differentiated) were cultured in the same medium without ascorbic acid. SW-403 (human colorectal cancer, established from a low differentiated tumour) and HepG2 (human hepatocellular carcinoma) cells were maintained in Dulbecco's MEM (Biochrom) with the same additives (except ascorbic acid) and under the same conditions. All cell lines were obtained from the German Collection of Microorganisms and Cell Cultures (Braunschweig, Germany). Cells were starved for 24 h in medium containing 0.125% FCS, trypsinized (0.05% Trypsin, 0.02% EDTA, Biochrom), seeded at a density of 0.5×10^6^ per well in six-well plates (9 cm^2^) (Becton Dickinson, Mannheim, Germany) or at a density of 5×10^3^ per well in 96-well plates (1 cm^2^) and incubated with 100, 200 or 500 μg ml^−1^ ciprofloxacin (concentration according to Aranha *et al*, 2000). Ciprofloxacin, which was friendly provided by Bayer (Leverkusen, Germany) was dissolved in water and further diluted in culture medium. If not otherwise mentioned, for collection of cells supernatants were saved after 18, 24, 48 or 72 h of incubation and centrifuged together with the trypsinized cells (1000 r.p.m. for 10 min). Further procession is described below.

### Flow cytometric analysis of apoptosis

Cell death was measured by lysing cells in a hypotonic solution containing 0.1% sodium citrate, 0.1% Triton X-100 and 50 μg ml^−1^ propidium iodide (Sigma, Deisenhofen, Germany) after two washes with PBS and Trypsin-EDTA solution. Analysis of the labelled nuclei was performed on a FACSCalibur fluorescence-activated cell sorter (FACS) using CELLQuest software (both from Becton Dickinson). The percentage of apoptotic cells was determined by measuring the fraction of nuclei that contained a sub-diploid DNA content. Ten thousand events were collected for each sample analyzed.

### BrdU-Incorporation ELISA

DNA-synthesis, which correlates well with cellular proliferation, was measured by bromodeoxyuridine (BrdU) incorporation using the Cell Proliferation ELISA (Roche Molecular Biochemicals, Mannheim, Germany) based on incorporation of BrdU into newly synthesized DNA and antibody-mediated detection of BrdU in DNA as described (Ruehl *et al*, 1999). Briefly, 5×10^3^ cells were seeded into 96-well microtiter plates (Falcon) and incubated with culture medium containing 10% FCS. BrdU was added to the cells together with CIP (10^−3^–10^−5^M). After 24 h cells were fixed and DNA denatured with an ethanolic solution (30 min), followed by incubation with an antibody to BrdU conjugated with peroxidase (60 min, 37°C). Immune complexes were detected using tetramethylbenzidine as substrate for 5 min, the reaction was stopped with H_2_SO_4_ and absorption measured at 450 nm in an ELISA reader (MRX II, Dynex, Frankfurt, Germany). The results are given as BrdU-incorporation (%) compared to untreated cells.

### Analysis of mitochondrial membrane potential

Mitochondrial injury was assessed by JC-1 staining (MoBiTec, Goettingen, Germany). This dye, existing as a monomer in solution emitting a green fluorescence, can assume a dimeric configuration emitting red fluorescence in a reaction driven by the mitochondrial transmembrane potential (Lawrence *et al*, 1993; Loeffler and Kroemer, 2000). Thus, red fluorescence of JC-1 indicates intact mitochondria, whereas green fluorescence shows monomeric JC-1 that remained unprocessed due to breakdown of the mitochondrial membrane potential (Reers *et al*, 1991). After trypsinization and centrifugation (RT, 10 min, 800 r.p.m.) the cell pellet resuspended in 1 ml medium, stained with 5 μg ml^−1^ JC-1 for 15 min at 37°C in the dark, then washed twice in PBS and resuspended in 0.5 ml PBS. Analysis was performed by FACS scan and mitochondrial function was assessed as JC-1 green (uncoupled mitochondria) or red (intact mitochondria) fluorescence (Smiley *et al*, 1991).

### Assessment of caspase activity

Caspase Colorimetric Assays (R&D Systems, Minneapolis, WI, USA) were used to determine the enzymatic activity of caspases 3, 8 and 9. The assays were performed according to the manufacturer's instructions after a 24 h incubation with increasing concentrations of ciprofloxacin. Caspase activation leads to the cleavage of the provided colorimetric substrates (all substrate peptides are conjugated to p-nitroaniline (pNA); caspase 3: DEVD-pNA, caspase 8: IETD-pNA, caspase 9: LEHD-pNA) and can be measured photometrically at 405 nm. According to the manufacturer and previous publications (Sanghavi *et al*, 1998; Dudich *et al*, 2000; Li *et al*, 2001) these amino acid sequences are the preferred ones of each caspase.

### Western blot analysis

Cells were lysed by adding 100 μl 2× sample buffer (2 mM NEM, 2 mM PMSF, 4% SDS, 4% DTT, 20% glycerol, 0.01% bromophenol blue, 2 M urea, 0.01 M Na-EDTA, 0.15 M Tris-HCl) to 10^6^ cells. DNA was sheared by pipetting up and down for 3 min at room temperature and suspensions were boiled at 95°C for 15 min, centrifuged at 13 000 r.p.m. for 10 min and subjected to 14% SDS–PAGE (pre-cast gels, Novex, San Diego, CA, USA). After blocking overnight at room temperature in a buffer containing PBS, 0.1% Tween 20 and 4% low fat milk powder, nitrocellulose membranes were incubated for 90 min either with polyclonal rabbit antibodies to human Bcl-2 (1 : 400, sc-783) or to human Bax (1 : 500, sc-493, both from Santa Cruz Biotechnology, Santa Cruz, CA, USA). Membranes were washed three times for 10 min in a buffer containing PBS, 0.1% Tween 20 and 4% low fat milk powder and incubated with a goat anti-rabbit IgG coupled to peroxidase (1 : 1000, Sigma, Deisenhofen, Germany) for 1 h at room temperature. Reactive bands were detected with the ECL chemiluminescence reagent (Amersham Pharmacia Biotech, Freiburg, Germany) and band intensities were analyzed by densitometry. Normalization was performed to β-actin.

### Statistical analysis

Statistical analysis was performed using SPSS for Windows (Release 9.0.0; SPSS Inc Chicago IL, USA). Significances were calculated using the *t*-test for paired samples *P*<0.05 was regarded as significant, *P*<0.01 as highly significant.

## RESULTS

### Morphological changes induced by ciprofloxacin

Figure 1

**Figure 1:**
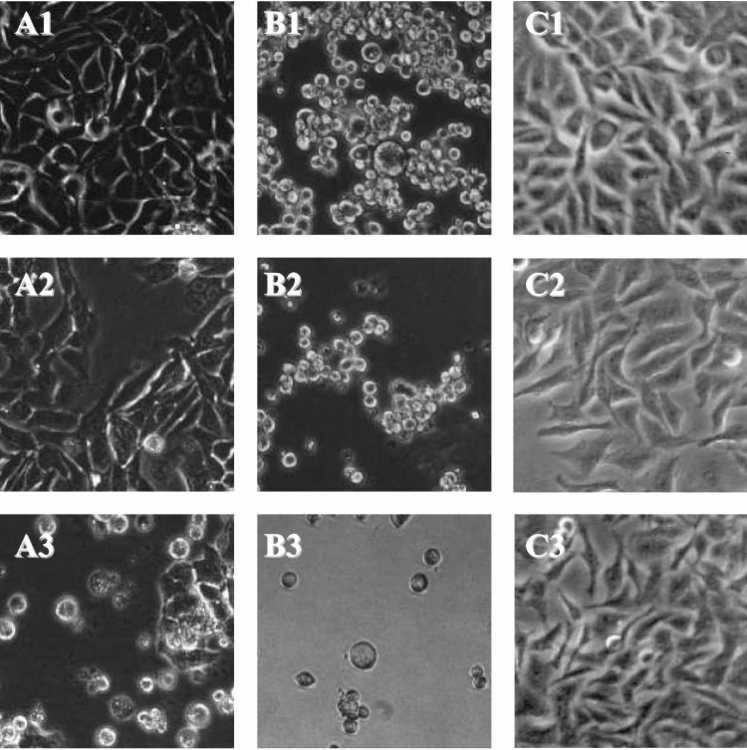
Ciprofloxacin induces morphological signs of apoptosis in colon cancer cell lines. Colon cancer cells CC-531 (**A**) and SW-403 (**B**) as well as hepatoma cells HepG2 (**C**) untreated (1) and after 18 h of incubation with 100 μg ml^−1^ (2) and 500 μg ml^−1^ (3) of ciprofloxacin.

shows morphological changes observed in CC-531, SW-403 and HT-29 cells after incubation with different concentrations of ciprofloxacin for 18 h. While untreated cells grew adherent on culture plates and were of a slender and spindle-shaped appearance, cells cultured with ciprofloxacin detached from their substratum, became rounded and pyknotic and showed apoptotic bodies, thus displaying the typical morphological changes for apoptotic cells. The control cell line HepG2 did not show comparable morphological changes.

### Ciprofloxacin inhibits colon carcinoma cell DNA synthesis

**Figure 2:**
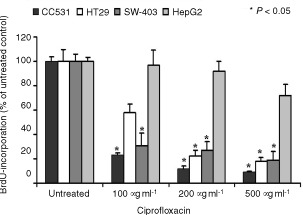
Ciprofloxacin inhibits DNA-synthesis in colon carcinoma cells. DNA-synthesis was measured by BrdU-incorporation in CC-531, HT-29, SW-403 and HepG2 hepatoma cells after treatment with 100, 200 or 500 μg ml^−1^ ciprofloxacin (24 h). Results for untreated cells were set at 100%. Values are means±s.d. of six independent experiments.

In CC-531 cells, incubation with ciprofloxacin at 100, 200 and 500 μg ml^−1^ for 24 h decreased the amount of BrdU incorporated into newly synthesized DNA from 100 to 23, 12 and 9%, respectively. Similar results were obtained for SW-403 and HT-29 cells. There was no antiproliferative effect of ciprofloxacin on HepG2 cells (Figure 2

).

### Ciprofloxacin induces cell death in human colon carcinoma cell lines via cell cycle arrest and mitochondrial membrane breakdown

**Figure 3:**
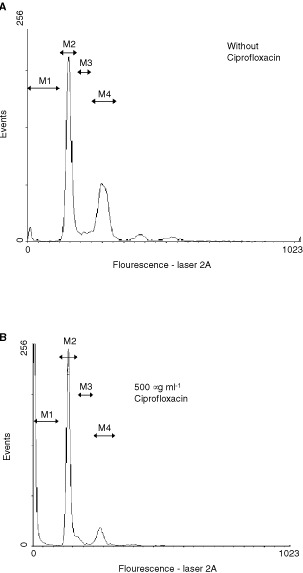
Apoptosis is induced after 24 h of incubation with ciprofloxacin. When compared to untreated control cells (**A**), incubation of SW-403 cells with 500 μg ml^−1^ (**B**) for 24 h caused a decrease of S/G_2_-cells (M3 and M4) and an increase of sub-G_1_-cells (M1) M2 marks cells in G1 phase.

As evidenced by FACS-analysis of the cell cycle, ciprofloxacin lead to a decrease of the G_2_-peak and an increase of sub-G_1_ events (correlates with apoptosis) (Figure 3

). Time- and dose-dependent induction of apoptosis were observed in CC-531 and HT-29 cells, while in SW-403 cells only doses of 200 and 500 μg ml^−1^ ciprofloxacin could induce significant rates of apoptosis after 18, 24, 48 and 72 h. In contrast, HepG2 cells remained largely unaffected (Table 1

**Table 1:**
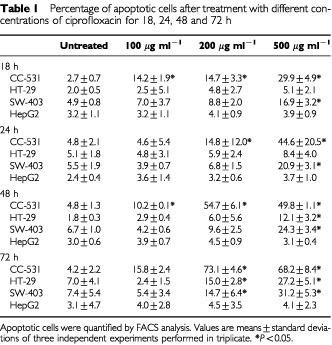
Percentage of apoptotic cells after treatment with different concentrations of ciprofloxacin for 18, 24, 48 and 72 h

, Figure 4

**Figure 4:**
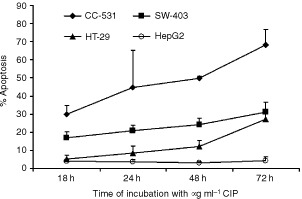
The Ciprofloxacin effect on apoptosis induction of colon cancer cells is time-dependent. Apoptosis rates were measured using FACS analysis. Results are given as means±s.d. of at least three independent experiments.

). While FACS analysis after staining with propidium iodide only visualizes the cell cycle, analysis after staining with JC-1 reveals the breakdown of mitochondrial membrane potential, which is known to be a key process during mitochondrial-dependent apoptosis. In fact, Figure 5

**Figure 5:**
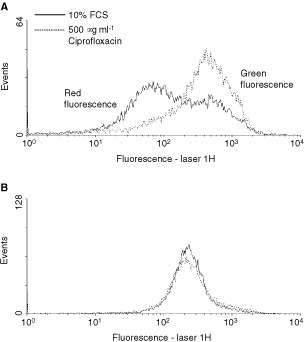
Ciprofloxacin induces mitochondrial injury of CC-531 (**A**) and HepG2 (**B**) cells. Green fluorescence of JC-1 dye was assessed as a parameter for mitochondrial breakdown. Increased green fluorescence is detectable in CC-531 cells incubated with 500 μg ml^−1^ ciprofloxacin for 48 h (dotted line) compared to cells treated with 10% FCS alone (black line) but not in HepG2 controls.

shows the increase in green *vs* red fluorescence, indicating increased mitochondrial breakdown in CC-531 but not HepG2 cells after 48 h of treatment with 500 μg ml^−1^ ciprofloxacin. For HT-29 and SW-403 cells similar data were obtained (not shown), while in HepG2 cells emitted fluorescence did not change after treatment with CIP (Figure 5). Experiments using different concentrations of CIP and different incubation periods showed fluorescent shift in a time- and dose-dependent manner.

### Ciprofloxacin activates caspases 3, 8 and 9

**Figure 6:**
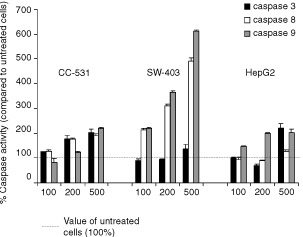
Different caspases are activated after incubation with ciprofloxacin. Activity of caspase 3, 8 and 9 was measured by a colourimetric peptide substrate-cleavage ELISA. Cells were treated with 100, 200 or 500 μg ml^−1^ ciprofloxacin (24 h). Basal activity of untreated cells was set at 100%. Values are means±s.d. of five independent experiments. Dotted line –––: value for untreated cells.

The basal activity of caspases was set to 100% in untreated cells and was measured after 24 h incubation with different concentrations of ciprofloxacin. The activity of caspase 3 increased significantly only in CC-531 cells after incuabtion with 200 or 500 μg ml^−1^ CIP, while no significant change was found in SW-403 cells and only after incubation with 500 μg ml^−1^ in HepG2 cells (Figure 6

). Activation of caspases 8 and 9 was dose-dependent, with highest values after incubation with 500 μg ml^−1^ CIP (in SW-403 cells). In CC-531 cells caspase-8 activity doubled after incubation with 200 and 500 μg ml^−1^, while in SW403 cell comparable results were induced even by 100 μg ml^−1^ CIP. Caspase 9 activity increased even more distinctly in SW-403 cells, while in CC-531 cells only the highest dose lead to significant caspase 9 activation. In HepG2 cells there was no change of caspase 8 activity, but doubled activation of caspase 9 after incubation with ⩾200 μg ml^−1^ (Figure 6).

### Pro-apoptotic Bax is upregulated by ciprofloxacin

**Figure 7:**
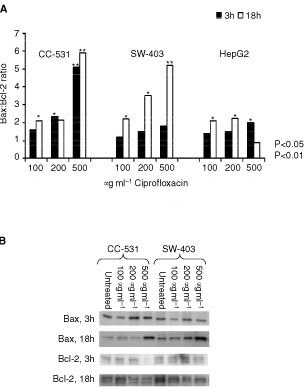
Ciprofloxacin increases the Bax : Bcl-2 ratio time-depently in colon cancer cells. Values of untreated cells were set to 1. Representative Western blot showing the increase of Bax and decrease of Bcl-2 expression in CC-531 and SW-403 cells.

Semi-quantitative Western blots were performed to investigate the effect of ciprofloxacin on pro-apoptotic Bax and anti-apoptotic Bcl-2. The ratio of Bax : Bcl-2 was set at 1.0 in untreated cells. Bax increased dose-dependently after 3 h of incubation in all tested cell lines, while Bcl-2 remained unchanged during the observation time. Whereas in CC-531 cells the Bax : Bcl-2 ratio increased to 1.6, 2.3 and 5.1 with 100, 200 and 500 μg ml^−1^ ciprofloxacin, respectively, only a minor response was found for SW-403 and HepG2 cells. After 18 h, however, the Bax : Bcl-2 ratio remained close to baseline in HepG2 cells, whereas it was highly increased in both CC cell lines (Figure 7

).

## DISCUSSION

We showed that exposure of CC-531, HT-29 and SW-403 colon carcinoma cells to ciprofloxacin caused a rapid suppression of *de novo* DNA synthesis. Furthermore, ciprofloxacin potently induced dose- and time-dependent apoptosis as shown by cell cycle analysis and breakdown of the mitochondrial potential. In contrast, ciprofloxacin had no or little effect on proliferation or apoptosis in the hepatoma cell line HepG2.

Although it is assumed that fluoroquinolones only inhibit bacterial type II DNA topoisomerase/gyrase, our results confirm that they can affect the growth of certain eukaryotic cells as well. We and others hypothesize, that these effects occur possibly via unselective inhibition of mitochondrial DNA-synthesis with subsequent mitochondrial injury (Lawrence *et al*, 1993). Thus, topoisomerase inhibitors might induce a selective loss of mitochondrial DNA, finally leading to depletion of intracellular ATP stores. Energy depletion favours apoptosis via induction of cell cycle arrest at the S/G_2_-M checkpoint, with concomitant down-regulation of cyclin B, cyclin E, dephosphorylation of cdk2, and an up-regulation of pro-apoptotic Bax (Jurgensmeier *et al*, 1998; Bratton *et al*, 2000). Therefore, we especially investigated the mitochondrial-dependent events during apoptosis, such as breakdown of mitochondrial membrane, expression of Bax and Bcl-2 and activation of caspase 9. In fact, we observed a breakdown of the mitochondrial membrane potential, which is most probably followed by release of cytochrome *c* into the cytosol, its interaction with CED-4 like protein Apaf-1 (Smiley *et al*, 1991; Jurgensmeier *et al*, 1998; Bratton *et al*, 2000). The CED-4-cytochrome complex activates pro-caspase 9 and initiates a proteolytic cascade finally leading to apoptosis (Smiley *et al*, 1991; Mancini *et al*, 1997; Jurgensmeier *et al*, 1998). Fluoroquinolone antibiotics may also trigger the Bax-pathway of apoptosis or interfere directly with mitochondrial membrane proteins, as described for cyclosporin A that binds to the mitochondrial megapore (Mancini *et al*, 1997; Jurgensmeier *et al*, 1998). Thus, activation of the mitochondrial permeability transition pore by Bax with subsequent release of cytochrome c has been described (Marzo *et al*, 1998, Narita *et al*, 1998). The ability of ciprofloxacin to activate Bax was recently shown in a bladder carcinoma cell line (Aranha *et al*, 2000). Here, we observed that ciprofloxacin mediates an up-regulation of Bax. Expression of Bcl-2, which remained unchanged in our experiments, inhibits apoptosis possibly via prevention of oxidative damage to subcellular components (Kroemer, 1997) and reduces caspase activity by preventing the formation of pro-apoptotic bodies (Zhang *et al*, 1999).

Caspase 3 is activated during the process of apoptosis and is one of the key enzymes required for the execution of the apoptotic programme. Caspase 8 (the major caspase to be activated by the TNF pathway) and caspase 9 (mitochondrial pathway) are initiator-caspases activating the downstream effector-caspases, especially caspase 3. Our results show significant changes of caspase 8 and 9 in all colorectal cancer cell lines, while caspase 3 was increased significantly only in CC-531 cells. All changes appeared to be dose-dependent. The observation of a well-balanced caspase activation profile in CC-531 cells and the distinctly higher activation of the initiator caspases *vs* caspase 3 in SW-403 cells appear to be related to a different progression of apoptosis. Thus, after 24 h of incubation with CIP, the apoptosis rates – as shown by FACS analysis – are higher in CC-531 than in SW-403 cells. Our results of an increase in caspase 8 and 9 demonstrate that CIP induces the membrane-related as well as the mitochondrial apoptosis pathway. Similarly, hypoxia can stimulate both apoptosis pathways in lymphoma cells (Malrhota *et al*, 2001). Our results of elevated bax and mitochondrial membrane potential breakdown further indicates the CIP may primarily mediate breakdown of mitochondrial membrane potential, while Caspase 8 is secondarily activated. However, this needs further evaluation by using caspase inhibitors.

In summary, we show that ciprofloxacin induces growth inhibition and apoptosis in colon carcinoma cell lines in a time- and dose-dependent manner, whereas hepatoma cells remained unaffected. The growth arrest is mediated through inhibition of DNA-synthesis, induction of mitochondrial injury and subsequent apoptosis. CIP can reach concentrations far above those of the serum in solid tissues, such as the lung. Therefore, due to the encouraging effects of this topoisomerase inhibitor CIP as well as other fluoroquinolones should be further investigated as (adjunctive) anti-tumoural agents in colorectal cancer cells.
